# Validation of the Orofacial Esthetic Scale in the general population

**DOI:** 10.1186/1477-7525-10-135

**Published:** 2012-11-19

**Authors:** Mike T John, Pernilla Larsson, Krister Nilner, Dipankar Bandyopadhyay, Thomas List

**Affiliations:** 1 Department of Diagnostic and Biological Sciences, School of Dentistry, University of Minnesota, Minneapolis, MN, USA; 2 Centre of Oral Rehabilitation, Linköping, Sweden; 3 Department of Oral Prosthetics, Faculty of Odontology, Malmö University, Malmö, Sweden; 4 Division of Biostatistics, School of Public Health, University of Minnesota, Minneapolis, MN, USA; 5 Department of Stomatognathic Physiology, Faculty of Odontology, Malmö University, Malmö, Sweden

## Abstract

**Background:**

The Orofacial Esthetic Scale (OES) is an eight-item instrument to assess how patients perceive their dental and facial esthetics. In this cross-sectional study we investigated dimensionality, reliability, and validity of OES scores in the adult general population in Sweden.

**Methods:**

In a random sample of the adult Swedish population (response rate: 39%, N=1159 subjects, 58% female, mean age (standard deviation): 49.2 (17.4) years), dimensionality of OES was investigated using factor analytic methods to determine how many scores are needed to characterize the construct. Reliability of scores was calculated using Cronbach’s alpha. Score validity was determined by correlating the OES summary score with a global indicator of orofacial esthetics (OE).

**Results:**

Factor analyses provided support that a single score can sufficiently characterize OE. A Cronbach’s alpha of 0.93 indicated excellent reliability. A validity coefficient of r=0.89 (95% confidence interval: 0.87-0.90) indicated that OES summary scores correlated highly with a global OE assessment.

**Conclusions:**

The OES is a promising instrument to measure the construct OE. Factor analyses supported that this construct can be assessed with one score, offering a feasible and acceptable standardized assessment of OE. The present study extends the OES use to the general population, an important target population for assessment of orofacial esthetics.

## Background

Orofacial esthetics is a major outcome of oral interventions. The appearance of the teeth, gums, and jaws is restored and changed by many restorative, periodontal, orthodontic and maxillofacial treatments.

To assess these treatment effects, the patient’s perspective is most important and questionnaires are needed for a standardized assessment. A newly developed questionnaire is the Orofacial Esthetic Scale [1,2]. The 8-item instrument was developed in Sweden, but an English version accompanied the original questionnaire. The dimensionality, reliability and validity of scores have been investigated in adult prosthodontic patients. With their various esthetical impairments, these patients represent an important target population; however, other target populations exist too, most notably the general population. Here, impairment assessment of orofacial esthetics is essential from a dental public health perspective. In addition, the general population is the source population for dental patients, i.e., patients with oral diseases arise from and return to this population after treatment. A validation of OES scores in the general population is therefore necessary and represents an important step in the psychometric evaluation of the scale. It was the aim of this study to investigate dimensionality, reliability, and validity of OES scores in the adult general population in Sweden.

## Methods

### Orofacial Esthetic Scale

The OES is a questionnaire that assesses orofacial esthetics. It was developed in prosthodontic patients, including reliability and validity assessment in this population [1,2]. The instrument contains eight items. Individuals are asked how they feel about the appearance of their face, mouth, teeth, and tooth replacements. They respond on a 0 to 10 numeric rating scale (0 - “very dissatisfied”, 10 - “very satisfied”) or mark the option “not applicable” if they don’t wish to respond. OES items refer to seven esthetic components (face, facial profile, mouth, rows of teeth, tooth shape/form, tooth color, gum). These seven items are combined into a summary score ranging from 0 to 70 (maximum score when patient is completely satisfied). An eighth OES item characterizes the patient’s global assessment of orofacial esthetics.

### Subjects

In a nationally representative random sample (N=3,000) of Swedish-speaking subjects, aged 18 years or older and drawn from the national population register (Folkbokföringen, a civil registry of Swedish inhabitants maintained by the Swedish Tax Agency), 1406 of the eligible subjects responded in a postal survey. OES questionnaires with 2 or less missing items were available for 1159 (39%) subjects. Missing OES data were imputed using median imputation. For details about socio-demographic and general health characteristics, missing data, as well as a non-response analysis see Larsson et al.[3]. The Regional Ethics Review Board at Linköping University Hospital reviewed and approved the study protocol. The project M208-07 “Munhälsa I Sverige” (Oral Health in Sweden) was approved on March 28th, 2008.

### Data analysis

#### Dimensionality

Our cross-sectional study investigated structural validity or factorial validity, which is a component of construct validity. According to Mokking et al., structural validity is “The degree to which the scores of an HR-PRO [health-related patient-reported outcome] instrument are an adequate reflection of the dimensionality of the construct to be measured” [4].

**Figure 1:**
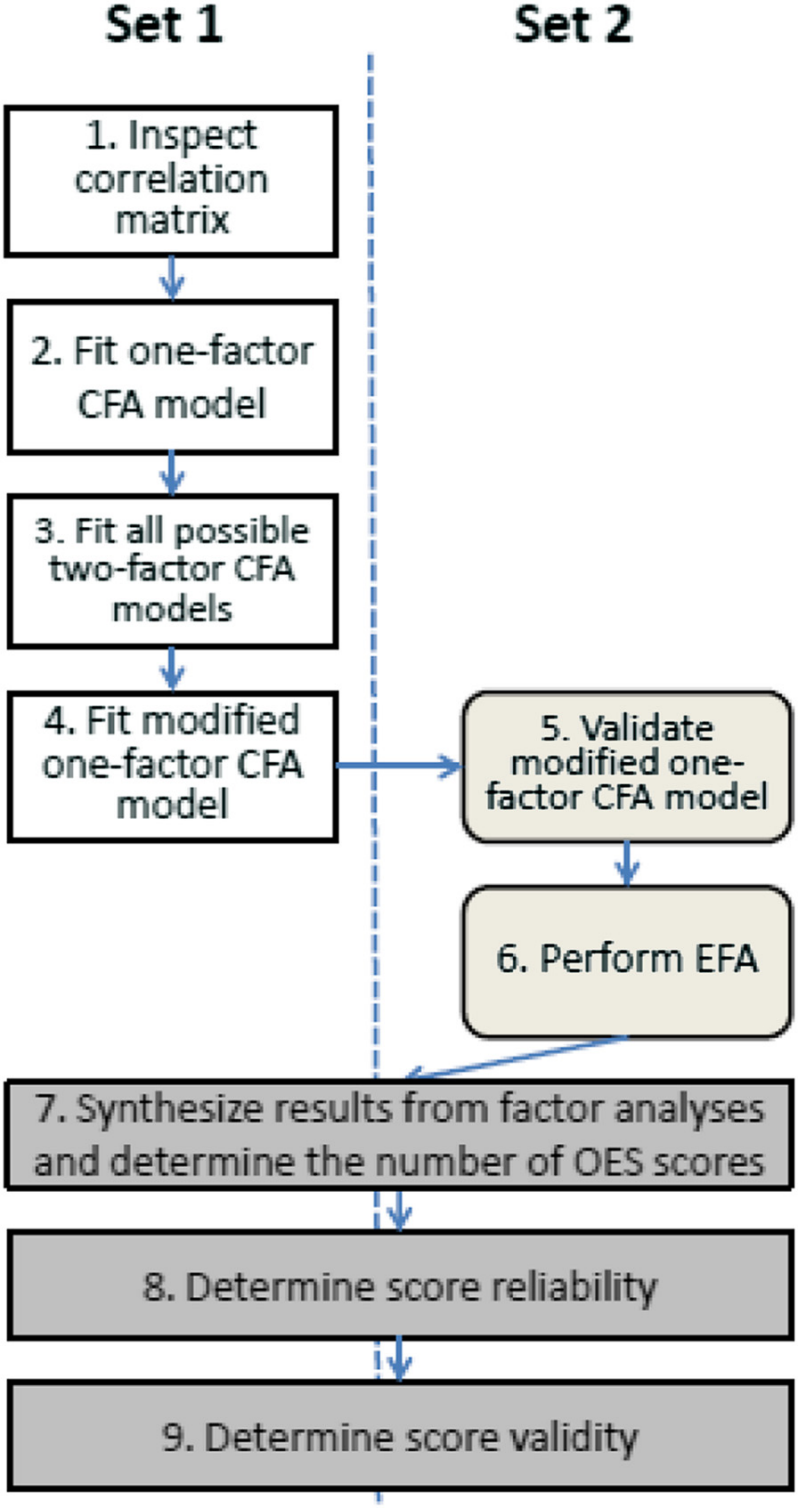
**Flow of dimensionality**, **reliability**, **and validity analyses in two random subsamples of the 1159 subjects.**

The analytical approach proceeded in a step-wise fashion. First, we split our data using computer generated random numbers (statistical software STATA version 12) into two random halves (“set 1” and “set 2”) of participants to decrease the number of analyses in one data set and to validate factor analyses (Figure 1). We inspected the correlation matrix of OES items (step 1). Based on the hypothesized unidimensional structure, we expected “moderate” to “strong” correlations (0.50-0.89 [5]) among items which should not vary substantially. Next, we fitted a one-factor model representing our unidimensionality hypothesis (step 2). However, we also considered all 35 possible two-factor models with 3-item and 4-item factors as alternatives and fitted them as well (step 3). As with other confirmatory factor analyses (CFA), the first OES item in each factor was used as marker indicator for the latent factor. To evaluate model fit, we used a set of indices suggested by Kline et al. [6]: chi-square test, standardized root mean square residual (SRMR), root mean square error of approximation (RMSEA), comparative fit index (CFI) and Tucker–Lewis index (TLI). Commonly applied guidelines for adequate model fit suggested [7]:

–SRMR: ≤0.08;

–RMSEA: ≤0.06 and models with RMSEA ≥0.1 should be rejected [8]; and

–CFI, TLI: ≥0.95.

After evaluating model fit for the one- and the two-factor models, we examined the residual matrix of the one-factor model to identify localized areas of strain. We considered differences between predicted and observed correlations of ≥0.10 as substantial [6] and also examined modification indices. Based on these results and using substantive knowledge, we modified the one-factor model, as this was our primary hypothesis for a factorial structure, and tested the modified model in the first data set again (step 4). The modified one-factor model was also tested in the second data set to validate findings in independent subjects (step 5). The existence of equivalent models, i.e., models that reproduce the same sets of corresponding covariance matrices but have different substantive interpretations [6], was explored. CFA analyses were performed with Stata 12 (StataCorp. 2011. Stata Statistical Software: Release 12. College Station, TX: StataCorp LP) using a maximum likelihood minimization function.

We also applied exploratory factor analysis (EFA) in step 6, in which the intent was to determine whether the type of factor analysis to identify the factors makes a difference. Using the principal factor method in the EFA, the number of factors was determined according to two criteria: the Kaiser criterion [9] and the scree plot [10]. Finally, we synthesized all factor analytic results and determined how many factors characterize the construct OE sufficiently (step 7).

#### Reliability

We determined internal consistency using Cronbach’s alpha [11], average inter-item correlation, and item-rest correlations.

#### Validity

We determined the correlation between the summary score of the seven OES items and the global assessment (eighth OES item) as a measure of score validity.

## Results

### Subject characteristics and severity of OES item impairment

The majority of subjects was female, between 32 and 66 years of age, and had at least a high school education (Table 1). About a third of subjects had only natural teeth, i.e., no partial and complete dentures. Esthetical impairment was moderate with mean scores of 6 to 7 on a 0–10 scale in which 10 indicates that subjects were very dissatisfied with their appearance. Splitting the sample into two sets did not result in any notable imbalance of socio-demographic characteristics or OES item severity.

**Table 1:** **Socio**-**demographic characteristics and OES item severity for all subjects combined and 2 random subsamples **(**set 1 and 2**)

	**All subjects*N****=****1159***	**Set 1*N****=****580***	**Set 2*N****=****579***
	**% (N**) **or mean**±**SD**		
**Female**	55.7 (645)	55.5 (322)	55.8 (323)
**Age** [**years**]^#^	49.2±17.4	49.3±17.4	49.0±17.4
**Elementary school**	21.8 (251)	21.4 (124)	22.2 (127)
**High school**	40.9 (471)	39.0 (226)	42.9 (245)
University degree*	37.3 (429)	39.7 (230)	34.9 (199)
**No partial or complete denture**^$^	33.7 (382)	33.6 (191)	33.7 (191)
**OES items** [**0** – **very dissatisfied**, **10** – **very satisfied with appearance**]			
**Face**	7.6±2.4	7.6±2.4	7.6±2.3
**Profile**	7.5±2.5	7.5±2.5	7.5±2.4
**Mouth**	7.2±2.7	7.2±2.7	7.2±2.8
**Alignment**	6.9±2.8	6.9±2.8	6.9±2.9
**Shape**	7.2±2.7	7.1±2.6	7.2±2.8
**Color**	6.3±2.9	6.2±3.0	6.4±2.8
**Gingiva**	7.6±2.4	7.6±2.4	7.6±2.4

Missing data for ^#^7, *8, and ^$^24 subjects.

### Dimensionality

#### Inspection of correlations among OES items

As expected, correlation coefficients varied between 0.52 and 0.87 in the first data set (Table 2). Standard errors of 0.03 and smaller indicated that estimates were precise. As expected, differences between two data sets’ correlation coefficients were small, except for one difference of 0.10. Inspection of the correlation matrix did not reveal an obvious pattern of correlation clusters and supported the hypothesis of a unidimensional construct.

**Table 2:** **Correlation matrix of OES items **(**lower triangle**: **Pearson correlation coefficients and standard errors for inter**-**item correlations in set 1**, **N**=**580**; **upper triangle**: **Differences between Pearson correlation coefficients between set 1 and 2**)

	**Face**	**Profile**	**Mouth**	**Alignment**	**Shape**	**Color**	**Gingiva**
**Face**	-	−0.02	0.01	−0.03	−0.04	−0.02	−0.01
**Profile**	0.86	-	0.00	−0.05	−0.04	−0.02	0.00
	(0.01)						
**Mouth**	0.76	0.69	-	−0.02	−0.02	0.00	−0.03
	(0.02)	(0.02)					
**Alignment**	0.67	0.60	0.85	-	−0.05	−0.03	−0.10
	(0.02)	(0.03)	(0.01)				
**Shape**	0.64	0.58	0.80	0.87	-	0.01	−0.04
	(0.02)	(0.03)	(0.02)	(0.01)			
**Color**	0.54	0.52	0.67	0.67	0.68	-	0.01
	(0.03)	(0.03)	(0.02)	(0.02)	(0.02)		
**Gingiva**	0.55	0.53	0.59	0.57	0.60	0.62	-
	0.03)	(0.03)	(0.03)	(0.03)	(0.03)	(0.03)	

#### Confirmatory factor analysis

**Figure 2:**
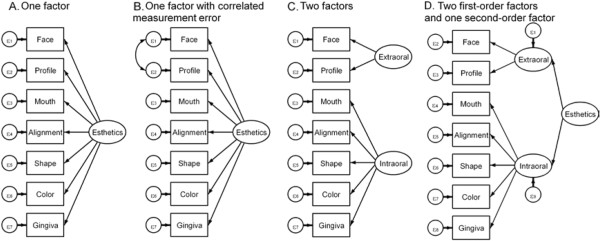
Hypothesized unidimensional factor structure tested in the first data set (A), modified unidimensional factor structure (B), and alternative factor structures equivalent with model B (C - Two-factor model, D - hierarchical model).

Model fit of the one-factor (unidimensional) model (Figure 2) reached an acceptable level only for the SRMR (Table 3). In general, two-factor models were better, but none of the 35 models reached acceptable levels for all 5 indices, indicating that these models did not provide desired model fit improvement over the one-factor model.

**Table 3:** **Fit statistics for a one**-**factor**, **35 two**-**factor and a modified one**-**factor confirmatory factor analysis model in set 1 and 2**

		**Set 1(N**=**580)**				**Set 2(N**=**579)**
	**one-factor model(df**=**14)**	**35 two-factor models(df**=**13)**			**modified one-factor modeldf**=**(13)**	**modified one-factor model(df**=**13)**
		**Median**	**IQR**^#^	**Range**		
Chi*	564.5	548.5	484.7-562.7	238.3-594.5	174.0	128.5
SRMR	0.06	0.06	0.06-0.06	0.05-0.09	0.04	0.03
RMSEA	0.26	0.27	0.25-0.27	0.17-0.28	0.15	0.12
CFI	0.85	0.85	0.85-0.87	0.84-0.94	0.96	0.97
TLI	0.77	0.76	0.76-0.79	0.74-0.90	0.93	0.95

^#^Interquartile range, *all models: P<0.001.

Inspection of the one-factor model’s matrix of residuals showed only two residuals of substantial magnitude, i.e., for the 21 OES item correlations, the one-factor model provided good fit for 19 correlations and not a good fit for 2 correlations. Specifically, the observed correlation between *(appearance of) face* and *profile* was 0.86 (Table 2), but the predicted correlation was only 0.58. The observed correlation between *color (of teeth)* and *(appearance of) gingiva* was 0.62 (Table 2), but the predicted correlation was only 0.50. Among the modification indices suggested by the software, a correlated measurement error for *face*-*profile* would result in the largest chi square improvement followed by a correlated measurement error for *color-gingiva*. We based our decision to modify the one-factor model on the magnitude of the residuals, the modification indices, AND substantive knowledge: *face* represents the frontal view and *profile* represents the lateral view of the extraoral appearance. Therefore, a modified one-factor model with a correlated measurement error between *face* and *profile* was created. This model provided better fit indices compared to all previous models. Only the RMSEA did not reach the suggested threshold value, but all correlation residuals were 0.07 and smaller, except for the above mentioned residual between *color* and *gums* of 0.12.

In the second data set, this model’s fit even improved slightly. Interpreting the model parameter, OES items’ factor loadings were of substantial magnitude and statistically significant (correlations’ range: 0.67-0.97). These loadings differed only marginally from the loadings in the one-factor model without correlated measurement error (all differences ≤0.06). Therefore, OES items seemed to be sound indicators of OE. In addition, model parameters did not differ notably between the one-factor models with and without correlated measurement error.

#### Exploratory factor analysis

In the exploratory factor analysis in data set 2, one factor with an eigenvalue >1 (Kaiser criterion) was found. The Screeplot supported the presence of one dominant latent factor.

#### Synthesis of visual inspection of the correlation matrix, confirmatory and exploratory factor analyses results

Visual inspection of the correlation matrix and EFA supported OE as a unidimensional construct. A unidimensional CFA model with a correlated measurement error between *face* and *profile,* equivalent to a two-factor or a hierarchical model (Figure 2), had the best model fit among several tested models. These results indicated that the construct OE can be adequately described with a single score.

#### Reliability

Cronbach’s alpha of 0.93 (lower limit of the 95% confidence interval: 0.93), average inter-item correlation of 0.67, and item-rest correlations ranging from 0.68 to 0.87 indicated satisfactory reliability.

#### Validity

The Pearson correlation coefficient between the seven-item summary score and the global assessment was high with r=0.89 (95% CI: 0.87 to 0.90).

## Discussion

Orofacial esthetics (OE) or appearance is a dimension of oral health-related quality of life [12], a comprehensive and important concept to characterize how individuals perceive their oral health, and it can be measured by the Orofacial Esthetics Scale (OES). This scale was originally developed in prosthodontics patients, but the present study extends the instrument’s use to the general population. For the adult general population, we provide evidence for the reliability and validity of OES scores that characterize the construct OE with a single summary score.

### Comparison with previous studies

The OES was recently recommended for assessment of esthetical concerns in prosthodontic patients, emphasizing that evaluation of psychometric properties such as structural validity is critical for health measurement scales in general [13]. In Swedish prosthodontic patients, OE was also found to be a unidimensional construct based on EFA [1]. Cronbach’s alpha was between 0.86 and 0.89 [2] and only slightly lower than 0.93 in this study. A study in Croatian prosthodontic patients showed alphas between 0.80 and 0.96 [14]. In Swedish patients, the validity coefficient was r=0.83 compared with r=0.89 in this study. Results of this study seem to be in line with previous studies despite a response rate of 39% in our present study which represents a notable potential for selection bias. This situation may have an influence on the dimensionality results if factors that influence participation in our study also influence OES dimensionality.

### Limitations and interpretation of dimensionality findings

Our dimensionality findings don’t agree completely with each other. Visual inspection of the correlation matrix (“intuitive factor analysis,” according to Gorsuch [15]) favored OES’ unidimensionality. EFA also supported unidimensionality according to several criteria. CFA findings were not so straightforward. The hypothesis of a unidimensional model was rejected by the chi-square test, and model fit indices were acceptable only for one out of the five selected measures.

How can this discrepancy between the EFA and CFA be explained when, conceptually, the two methods should lead to the same conclusions? The two methods differ in their criteria for what is adequate model fit. For EFA, the substantial first latent factor and the substantial eigenvalue differences between the first and subsequent latent factors (Kaiser criterion, Screeplot) were sufficient to view OE as unidimensional. The CFA applies different criteria. The chi-square test rejected unidimensionality. This is not too surprising because this test is sensitive to sample size. For models with more than 400 subjects (we analyzed 579 and 580 subjects in the two sets), the chi-square statistic is almost always statistically significant [16]. When exploring the SRMR, the only fit index that does not include the chi-square value, a different picture emerged. Conceptually, the SRMR represents the average discrepancy between the correlations observed in the sample correlation matrix and the model-predicted correlations. The SRMR was between 0.03 and 0.06 for all models. In our opinion, this is small in absolute and relative magnitude (taking the average inter-item correlation of 0.66 into account). On average, discrepancies between observed and predicted correlations were reasonable. In addition, individual residuals were by and large acceptable. Assessing individual residuals to detect “localized areas of strain” is commonly recommended [17]. It was also recommended that fit indices should not even be computed for small degree of freedom models (such as ours), but rather the source of specification error should be identified [16]. We followed that recommendation and identified only two fitted residuals out of the 21 correlations that were larger than 0.10 – a rule of thumb recommended for adequate fit in the SEM literature [6].

That CFA is unable to confirm EFA results has been observed before [18,19] and it has been pointed out that the two techniques are not fully comparable [20], e.g., in their criteria to evaluate models as we discussed above. In our data, findings were only slightly different across methods. The strong latent factor was sufficient for EFA to view OE as unidimensional, whereas the CFA viewed the items *face* and *profile* as indicators for a second factor worthwhile to be identified for increased model fit. However, statistical significance is different from clinical relevance and the last step of a CFA – to consider equivalent models – provides interesting insight into the construct OE. Equivalent models have identical goodness of fit but different substantive interpretations [21]. Among several equivalent models, we considered a two-factor model (model C, Figure 2) and a hierarchical model (model D, Figure 2) as important alternatives. This two-factor model is different compared to the 35 two-factor models we investigated in the first data set. This model has only two items for the second latent factor, which is the minimum for identification [6], compared to three indicators we used for more robust factor identification according to recommendations [22]. The interpretation of this model, and also the hierarchical model which just adds a second-order factor summarizing the OE construct, is that OE may have an extraoral and an intraoral component. This seems plausible. Facial (=extraoral) and dental (=intraoral) esthetics are well-known terms in dentistry representing these concepts. For example, facial and dental appearances were distinguished in patients with bilateral cleft lip and palate [23]. Another study showed that esthetic dental and facial measurements were important factors for patient satisfaction and should be considered in esthetic anterior oral rehabilitation [24].

Summarizing all factor analytic results, the reliability as well as the validity findings, we recommend a simple characterization of the construct OE with one summary score. While we have not investigated other types of validity and reliability that could also be informative about the dimensionality of OEs, at this moment, we don’t consider the possible distinction between intra- and extraoral esthetics as worthwhile to be described by two scores. However, we believe that future studies should explore this further.

## Conclusions

The OES is a promising instrument to assess OE. Factor analyses supported that this construct can be characterized with one score. In addition, the present study extends the instrument’s use to the general population, an important target population.

## Competing interests

The authors declare that they have no competing interests.

## Authors’ contributions

MTJ, PL, KN, and TL conceptualized the rationale and designed the study. PL, KN, and TL contributed to the collection of data. MTJ and DB contributed to the statistical analysis and interpretation of the data. MTJ drafted the manuscript. PL, KN, DB, and TL revised the manuscript. All authors read and approved this study.
